# Incidence and Outcomes of Nontraumatic Shock in Adults Using Emergency Medical Services in Victoria, Australia

**DOI:** 10.1001/jamanetworkopen.2021.45179

**Published:** 2022-01-26

**Authors:** Jason E. Bloom, Emily Andrew, Luke P. Dawson, Ziad Nehme, Michael Stephenson, David Anderson, Himawan Fernando, Samer Noaman, Shelley Cox, Catherine Milne, William Chan, David M. Kaye, Karen Smith, Dion Stub

**Affiliations:** 1 Department of Cardiology, Alfred Health, Melbourne, Victoria, Australia; 2 Baker Heart and Diabetes Institute, Melbourne, Victoria, Australia; 3 Department of Cardiology, Western Health, St Albans, Victoria, Australia; 4 Ambulance Victoria, Blackburn, Victoria, Australia; 5 School of Public Health and Preventive Medicine, Monash University, Melbourne, Victoria, Australia; 6 Department of Paramedicine, Monash University, Frankston, Victoria, Australia

## Abstract

**Importance:**

Nontraumatic shock is a challenging clinical condition, presenting urgent and unique demands in the prehospital setting. There is a paucity of data assessing its incidence, etiology, and clinical outcomes.

**Objective:**

To assess the incidence, etiology, and clinical outcomes of patients treated by emergency medical services (EMS) with nontraumatic shock using a large population-based sample.

**Design, Setting, and Participants:**

This population-based cohort study included consecutive adult patients with shock not related to trauma who received care by EMS between January 1, 2015, and June 30, 2019, in Victoria, Australia. Data were obtained from individually linked ambulance, hospital, and state death index data sets. During the study period there were 2 485 311 cases attended by EMS, of which 16 827 met the study’s inclusion criteria for shock.

**Main Outcomes and Measures:**

The primary outcome was 30-day mortality. Secondary outcomes included length of hospital stay, emergency department discharge disposition, rates of coronary angiography and revascularization procedures, and the use of mechanical circulatory support.

**Results:**

A total of 12 695 patients were successfully linked, with a mean (SD) age of 65.7 (19.1) years; 6411 (50.5%) were men. The overall population-wide incidence of EMS-treated prehospital shock was 76 (95% CI, 75-77) per 100 000 person-years. An increased incidence was observed in men (79 [77-81] per 100 000 person-years), older patients (eg, aged 70-79 years: 177 [171-183] per 100 000 person-years), regional locations (outer regional or remote: 100 [94-107] per 100 000 person-years), and in areas with increased socioeconomic disadvantage (lowest socioeconomic status quintile: 92 [89-95] per 100 000 person-years). Patients with hospital outcome data were stratified into shock etiologies; 3615 (28.5%) had cardiogenic shock: 3998 (31.5%), septic shock; 1457 (11.5%), hypovolemic shock; and 3625 (28.6%), other causes of shock. Nearly one-third of patients (4158 [32.8%]) were deceased at 30 days. In multivariable analyses, increased age (all etiologies: hazard ratio [HR], 1.04; 95% CI, 1.03-1.04), female sex (cardiogenic shock: HR, 1.26; 95% CI, 1.12-1.42), increased initial heart rate (all etiologies: 1.01; 95% CI, 1.00-1.01), prehospital intubation (all etiologies: HR, 3.93; 95% CI, 3.48-4.44), and preexisting comorbidities (eg, chronic kidney disease, all etiologies: HR, 1.25; 95% CI, 1.10-1.42) were independently associated with 30-day mortality, while higher socioeconomic status (all etiologies: HR, 0.96; 95% CI, 0.94-0.98) and increased initial systolic blood pressure (all etiologies: HR, 0.99; 95% CI, 0.99-0.99) were associated with lower risk.

**Conclusions and Relevance:**

This population-level cohort study found that EMS-treated nontraumatic shock was a common condition, with a high risk of morbidity and mortality regardless of etiology. It disproportionately affected men, older patients, patients in regional areas, and those with social disadvantage. Further studies are required to assess how current systems of care can be optimized to improve outcomes.

## Key Points

**Question:**

What are the incidence and clinical outcomes of adults with shock that is not related to trauma who accessed the emergency medical service (EMS) system in Victoria, Australia?

**Findings:**

In this cohort study, the incidence of prehospital shock treated by EMS was 76 per 100 000 person-years, and the 30-day mortality rate was 32.8%.

**Meaning:**

These findings suggest that nontraumatic shock of varying etiologies among adults initially receiving care from EMS is associated with a high risk of morbidity and mortality.

## Teaser

This population-based cohort study assesses the incidence, etiology, and clinical outcomes of patients with nontraumatic shock treated by emergency medical services (EMS) in Victoria, Australia.

## Introduction

Shock is a clinical syndrome that is characterized by cellular and tissue hypoxia due to either inadequate oxygen delivery, increased oxygen demand, or a combination of these processes.^1,2,3^ Patients present on a spectrum of illness severity, ranging from occult hypoperfusion (with preserved blood pressure) to fulminant circulatory collapse.^1,2^ The altered physiology observed in shock can be broadly attributed to 4 mechanisms: hypovolemia, cardiogenic, obstructive, or distributive causes. Enhanced in-hospital systems of care, with effective resuscitation and supportive measures, early antibiotic administration, and cardiac reperfusion therapies, have resulted in incremental improvements in clinical outcomes.^4,5,6,7,8^ However, despite these improvements, shock remains a clinical condition that has a reported short-term mortality rate ranging from 20% to 50%.^7,9^

The management of shock within the hospital environment is an increasingly well characterized entity with a rapidly expanding body of trial and observational data.^6,10^ However, in the prehospital setting, there remains a paucity of contemporary clinical and epidemiologic data relating to the burden of this clinical syndrome. Using a large Australian emergency medical services (EMS) database, which has been linked to health service medical records and death index data, we aimed to define the incidence, etiologies, and clinical outcomes of nontraumatic shock among patients treated by EMS.

## Methods

This study was conducted and reported in accordance with the Strengthening the Reporting of Observational Studies in Epidemiology (STROBE) guidelines.^6^ Ethics approval for the data linkage, in addition to this specific analysis, was granted by the Monash University Human Research Ethics Committee. The requirement for informed consent was waived due to deidentified data being used in the analysis.

### Study Design and Participants

This population-based cohort study included consecutive adult patients attended to by EMS presenting with shock between January 1, 2015 and June 30, 2019, in Victoria, Australia, a state of 6.7 million people with a land area of 227 444 km^2^ that is located on the southeastern seaboard of the country. Within Victoria, approximately 70% of the population lives in the greater Melbourne metropolitan region, and the remaining population within semiurban and rural areas. Paramedic electronic patient care record data (ePCR) were linked to the Victorian Emergency Minimum Data set (VEMD), the Victorian Admitted Episodes Data set (VAED), Victorian Ambulance Cardiac Arrest Registry (VACAR), and the Victorian Death Index (VDI).

### Data Sources and Setting

Ambulance Victoria is the sole provider of emergency medical services in the state of Victoria, Australia. Access to EMS is provided through a single nationwide telephone number (ie, triple zero or 000). The EMS is funded primarily through the Victorian State Government, with a proportion of funding generated through membership subscription and transport fees.^11^ The EMS system provides a 2-tiered response to medical emergencies in the community: (1) approximately 4000 advanced life support paramedics who are capable of laryngeal mask airway insertion and medication administration (ie, analgesics, bronchodilators, and aspirin) and (2) approximately 500 intensive care paramedics capable of endotracheal intubation and a wider scope of medications (including intravenous epinephrine infusions and thrombolytics), who are dispatched for emergencies that include cardiac arrest and suspected acute coronary syndrome. Epinephrine is currently the only vasopressor carried by EMS in Victoria and is protocolized for any shock, regardless of suspected etiology. At the conclusion of each case, paramedics complete an ePCR that captures patient and case details, preexisting comorbidities, and any management provided. Data from these records are uploaded to and stored within a clinical data warehouse and are available for analysis.

For this study, data linkage was performed to combine ePCR data with key data sets. These included VEMD, a Victorian Department of Health administrative and clinical data set including all emergency department (ED) presentations at public hospitals in the state; VAED, a Victorian Department of Health demographic, clinical, and administrative data set detailing each admitted episode of care occurring in public and private hospitals, as well as rehabilitation centers, extended care facilities, and day procedure centers in the state; VACAR, a clinical quality registry that records details of all out-of-hospital cardiac arrest events where EMS attend^11,12^; and VDI, a Victorian Department of Health data set that captures the date and cause of all deaths in Victoria. A detailed description of the linkage processes and methodologies employed are described in eMethods in the Supplement.

### Study Definitions

Shock was defined as sustained hypotension (systolic blood pressure <90 mm Hg sustained for >30 minutes) or EMS-administered epinephrine (either as an intravenous bolus or infusion). Exclusion criteria included known traumatic etiology, deceased at scene, for palliative treatment only, ambulance attendances for transfers between hospitals, and younger than 18 years. Final diagnosis was defined according to *International Classification of Diseases and Related Health Problems, Tenth Revision (ICD-10)* coding as the VAED primary diagnosis if discharged from hospital or the VEMD primary diagnosis if discharged from the ED. Patients were included in the primary analysis if they had a successful linkage to VAED or VEMD. Using the primary *ICD-10* diagnosis, linked patients were stratified into 4 shock etiology groups: (1) cardiogenic shock; (2) septic shock; (3) hypovolemic shock; and (4) other cause of shock (including obstructive causes). Further details relating to the *ICD-10* diagnostic codes used to classify shock etiology are described in the eMethods in the Supplement.

### Outcomes

The primary end point was 30-day mortality. Secondary outcomes reported include length of hospital stay, ED discharge disposition, rates of coronary angiography and revascularization procedures, and the use of mechanical circulatory support.

### Statistical Analysis

Incidence rates per 100 000 person-years were calculated using midyear age and sex–specific population-wide estimates for person-years at risk available from the Australian Bureau of Statistics and using the total number of patients meeting the study’s inclusion criteria (eFigure 1 in the Supplement). We derived 95% CIs for incidence rates with the assumption that the observed number of episodes followed a Poisson distribution, and trend was assessed for significance using the Cochran-Armitage test. Patient characteristics for the successfully linked cohort were presented as a total and stratified according to shock etiology. Continuous variables are presented as medians and IQRs or mean and SD (as appropriate). Categorical variables are presented as frequencies and percentages. All tests were 2-tailed and assessed at the 5% significance level. Comparisons of continuous variables were performed using *t* tests and one-way analysis of variance for normally distributed data or the Mann-Whitney U or Kruskal-Wallis test for nonparametric variables. Differences in proportions were assessed using the χ^2^ test. Unadjusted and adjusted (for age and sex) hazard ratios (HRs) for 30-day mortality for shock of various etiologies were derived using Cox regression models, with other shock used as the reference. Association between prehospital covariates and mortality were assessed through Cox regression models performed for the entire matched cohort and individual etiologies of shock, censored at 30 days. Clinically relevant covariates were selected for inclusion in the models and comprised age, sex, prior history of diabetes, coronary artery disease, hypertension, prehospital cardiac arrest (for the cardiogenic shock group), chronic kidney disease (CKD), heart failure, stroke (cerebrovascular accident [CVA]), peripheral vascular disease (PVD), obstructive airways disease (chronic obstructive pulmonary disease [COPD]), initial blood pressure and heart rate, prehospital intubation, use of an epinephrine infusion, remoteness, and socioeconomic status. Statistical analysis was conducted using Stata version 16.1 for Windows (StataCorp).

## Results

Between January 2015 and June 2019 (equating to 22 186 930 person-years), 16 827 patients met the study’s inclusion criteria, with a mean (SD) age of 66.3 (19.0) years; 8555 (50.9%) were men (eFigure 1 in the Supplement). There was a crude incidence rate of 6.8 per 1000 EMS attendances. Of the 20 890 patients with prehospital hypoperfusion who either met the exclusion criteria or were not successfully transported to hospital, 4163 (19.9%) died at the scene or during transport, 60 (0.3%) received palliative management at the scene, 289 (1.4%) refused transport, and 806 (3.9%) were not transported for other reasons (eFigure 1 and eTable 1 in the Supplement), resulting in a total of 15 572 patients eligible for linkage with VAED or VEMD. Of those who survived to hospital, successful linkage was achieved in 12 695 patients (81.5%). The final linked cohort included 3615 (28.5%) cardiogenic shock cases, 3998 (31.5%) septic shock cases, 1457 (11.5%) hypovolemic shock cases, and 3625 (28.6%) cases of shock with other causes.

The incidence of EMS attendance for all-cause shock was 76 (95% CI, 75-77) per 100 000 person years and is presented in Table 1. There was a greater incidence observed in men compared with women (79 [77-81] per 100 000 person-years), in regional locations (outer regional or remote: 100 [94-107] per 100 000 person-years), among those with increased socioeconomic disadvantage (lowest socioeconomic status quintile: 92 [89-95] per 100 000 person-years), and a stepwise increase in incidence across more advanced age group strata (eg, aged 70-79 years: 177 [171-183] per 100 000 person-years). A temporal reduction in the overall incidence of shock over the study period was demonstrated with 94 per 100 000 person years observed in 2015 compared with 67 per 100 000 person years in 2019 (*P* for trend < .001) (eTable 2 in the Supplement).

**Table 1.** Incidence of Ambulance Attendances for Shock According to Sex, Age, Region, and Socioeconomic Status

Characteristic	Incidence (95% CI), per 100 000 person-years^a^
Overall	76 (75-77)
Sex	
Male	79 (77-81)
Female	73 (71-75)
Age, y	
18-29	21 (20-22)
30-39	25 (24-27)
40-49	43 (41-45)
50-59	62 (59-64)
60-69	104 (100-108)
70-79	177 (171-183)
≥80	414 (402-426)
Region	
City	68 (67-70)
Inner regional	90 (87-93)
Outer regional or remote	100 (94-107)
Socioeconomic status quintile	
1, lowest	92 (89-95)
2	72 (70-75)
3	64 (62-66)
4	58 (56-69)
5, highest	46 (44-48)

^a^ The 95% CIs were derived with the assumption that the observed number of episodes followed a Poisson distribution.

### Baseline Characteristics and EMS Interventions

The linked cohort had a mean (SD) age of 65.7 (19.1) years and included 6411 (50.5%) men (Table 2). The cardiogenic and septic shock groups were older compared with the hypovolemic and other causes of shock groups. Those with cardiogenic shock had increased rates of traditional cardiovascular risk factors that included hypertension, dyslipidemia and diabetes, in addition to preexisting coronary artery disease and heart failure. Using the VACAR, within the cardiogenic shock group, 1518 (42.0%) experienced prehospital cardiac arrest. A sensitivity analysis was performed comparing baseline characteristics and mortality outcomes in cardiogenic shock with and without prehospital cardiac arrest (eTable 3, eTable 4, and eFigure 2 in the Supplement). There were no differences observed between the shock etiology groups with respect to rates of geographic remoteness and socioeconomic disadvantage.

**Table 2.** Baseline Characteristics of Sample

Characteristic	Patients by shock etiology, No. (%)					*P* value
	All (N = 12 695)	Cardiogenic shock (n = 3615)	Septic shock (n = 3998)	Hypovolemic shock (n = 1457)	Other causes of shock (n = 3625)	
Age, mean (SD), y	65.7 (19.1)	68.3 (16.1)	69.8 (17.7)	65.3 (19.6)	58.6 (21.2)	<.001
Male	6411 (50.5)	2160 (59.8)	1940 (48.5)	682 (46.8)	1629 (44.9)	<.001
Female	6284 (49.5)	1455 (40.2)	2058 (51.5)	775 (53.2)	1996 (55.1)	<.001
Accessibility and Remoteness Index of Australia						
Major cities of Australia	9147 (73.3)	2600 (73.2)	2887 (72.6)	1085 (75.2)	2575 (73.4)	.60
Inner regional Australia	2706 (21.7)	783 (22.0)	883 (22.2)	288 (20.0)	752 (21.4)	
Outer regional Australia	629 (5.0)	171 (4.8)	206 (5.2)	70 (4.9)	182 (5.2)	
Index of Relative Socioeconomic Disadvantage						
1, most disadvantaged	3063 (27.2)	865 (26.8)	997 (27.9)	354 (27.6)	847 (26.8)	.48
2	2449 (21.8)	690 (21.9)	784 (21.9)	301 (23.5)	674 (21.3)	
3	2187 (19.4)	633 (19.6)	704 (19.7)	238 (18.6)	612 (19.3)	
4	1975 (17.6)	561 (17.4)	625 (17.5)	207 (16.2)	582 (18.4)	
5, least disadvantaged	1577 (14.0)	481 (14.9)	464 (13.0)	181 (14.1)	451 (14.2)	
Preexisting medical conditions						
Hypertension	3927 (33.2)	1348 (41.5)	1246 (32.0)	433 (31.4)	900 (27.1)	<.001
Dyslipidemia	2301 (19.4)	841 (25.9)	705 (18.1)	248 (18.0)	507 (15.3)	<.001
Diabetes	2165 (18.3)	709 (21.8)	737 (18.9)	303 (22.0)	416 (12.5)	<.001
Coronary artery disease	2154 (18.2)	835 (25.7)	667 (17.1)	222 (16.1)	430 (12.9)	<.001
Cardiac failure	1286 (10.9)	439 (13.5)	490 (12.6)	115 (8.4)	242 (7.3)	<.001
Chronic kidney disease	731 (6.2)	223 (6.9)	272 (7.0)	95 (6.9)	141 (4.2)	<.001
Peripheral vascular disease	187 (1.6)	52 (1.6)	76 (2.0)	24 (1.7)	35 (1.1)	.02
Cerebrovascular disease	908 (7.7)	240 (7.4)	345 (8.9)	99 (7.2)	224 (6.7)	<.001
Airways disease	1209 (10.2)	320 (9.9)	577 (14.8)	97 (7.0)	215 (6.5)	<.001
Initial systolic blood pressure, median (IQR), mm Hg^a^	70 (0-80)	58 (0-80)	75 (60-80)	70 (55-80)	70 (0-80)	<.001
Initial heart rate, median (IQR), beats/min	90 (70-120)	84 (50-124)	100 (80-120)	90 (74-112)	86 (68-110)	<.001
Initial temperature, mean (SD), °C	36.2 (1.6)	35.8 (1.4)	36.8 (1.7)	35.9 (1.7)	36.1 (1.6)	<.001
Initial oxygen saturation, mean (SD) %	90.4 (12.7)	89.2 (16.7)	89.2 (10.9)	93.5 (8.5)	91.9 (10.6)	<.001
Initial respirator rate, median (IQR), breaths/min	18 (16-24)	16 (0-20)	22 (18-32)	18 (16-24)	16 (16-20)	<.001
Intensive care paramedic attendance	5960 (46.9)	2460 (68.0)	1529 (39.8)	496 (34.0)	1457 (40.2)	<.001
Time at scene, median (IQR), min	30 (20-46)	41 (25-60)	27 (20-39)	26 (18-36)	27 (17-42)	<.001
Transport time, median (IQR), min	18 (11-28)	18 (11.7-27)	19 (12-29)	18 (11-29)	18 (11-28)	.11
Prehospital intubation	1878 (14.8)	1385 (38.3)	100 (2.5)	20 (1.4)	373 (10.3)	<.001
Prehospital cardiac arrest requiring CPR^b^	1906 (15)	1518 (42)	67 (1.7)	20 (1.4)	301 (8.3)	<.001
Epinephrine infusion commenced	3845 (30.3)	1960 (54.2)	903 (22.5)	113 (7.8)	869 (24)	<.001
Maximum epinephrine infusion, mean (SD), µg/min	30.1 (48.7)	40.9 (57.0)	12.6 (18.7)	18.5 (35.8)	23.5 (41.0)	<.001
Epinephrine infusion, mean (SD) µg/min	21.8 (33.9)	29.3 (39.9)	9.6 (12.4)	14.1 (25.2)	18.7 (30.9)	<.001

Abbreviation: CPR, cardiopulmonary resuscitation.

^a^ Undetectable initial blood pressure was recorded as 0 mm Hg.

^b^ Determined from Victorian Ambulance Cardiac Arrest registry.

The overall linked cohort was hypotensive, with a median (IQR) initial systolic blood pressure of 70 [IQR 0-80] mm Hg (Table 2). Those with cardiogenic shock were significantly more hypotensive compared with the other shock etiologies (median (IQR) systolic blood pressure: cardiogenic: 58 [0-80] mm Hg; septic, 75 [60-80] mm Hg; hypovolemic, 70 [55-80] mm Hg; other, 70 [0-80] mm Hg; *P* < .001). These findings correspond with 1960 patients with cardiogenic shock (54.2%) receiving an epinephrine infusion, compared with 903 patients with septic shock (22.5%), 113 patients with hypovolemic shock (7.8%), and 869 patients with other causes of shock (24.0%) (*P* < .001). Furthermore, the maximum infusion rate and average infusion dose of epinephrine was significantly higher in those with cardiogenic shock. Prehospital intubation also occurred significantly more frequently in those with cardiogenic shock who experienced prehospital cardiac arrest (cardiogenic shock with prehospital cardiac arrest, 1316 of 1518 [86.7%]; cardiogenic shock without prehospital cardiac arrest, 69 of 2097 [3.3%]; septic shock, 100 of 2998 [2.5%]; hypovolemic shock, 20 of 1457 [1.4%]; and other causes of shock, 373 of 3625 [10.3%]; *P* < .001). Transport times were similar among groups, with a median (IQR) of 18 (IQR 11-28) minutes. However, the median (IQR) time spent at the scene was significantly longer for the cardiogenic shock without cardiac arrest group (29 [19-43] minutes; septic, 27 [20-39] minutes; hypovolemic, 26 [18-36] minutes; other, 27 [14-42] minutes; *P* < .001). Among patients with cardiogenic shock, those who experienced prehospital cardiac arrest had significantly longer median (IQR) time spent at the scene by EMS compared with those without cardiac arrest (58 [45-76] minutes vs 29 [19-43] minutes; *P* < .001)

A total of 1168 patients (9.2%) died within 24 hours of arrival to hospital, resulting in an overall median (IQR) hospital length of stay of 2 (1-10) days. Median (IQR) length of stay was significantly longer for septic shock (4 [1-8] days) and cardiogenic shock (3 [1-7] days) compared with hypovolemic shock (2 [1-5] days) and other etiologies (2 [1-10] days) (*P* < .001). Patients were admitted to the intensive care unit (ICU) directly from the ED in 19.3% of cases, with a median (IQR) ICU length of stay of 59 (25-114) hours. Overall, 168 patients with cardiogenic shock (5.2%) were transferred to another hospital from the original receiving ED. Of those that survived to 30 days, length of hospital stay was a median (IQR) of 4 (1-9) days.

Among patients with cardiogenic shock, 979 (27.1%) underwent coronary angiography during their inpatient stay (493 [13.6%] received percutaneous coronary intervention and 21 [0.6%] coronary artery bypass grafting as a mode of revascularization). Rates of inpatient coronary angiography were significantly higher in cardiogenic shock with cardiac arrest compared with those without cardiac arrest (605 [39.9%] vs 374 [17.9%]; *P* < .001). Only 95 patients (0.8%) from the matched cohort received mechanical circulatory support (either extracorporeal membrane oxygenation or intra-aortic balloon pump).

### Outcomes

Of the 12 695 successfully linked patients, 4158 deaths (32.8%) occurred by 30 days (Table 3). The 30-day mortality rate was highest in those with cardiogenic shock (1563 deaths [43.2%]), followed by septic shock (1520 deaths [38.0%]), hypovolemic shock (416 deaths [28.6%]), and other causes of shock (659 deaths [18.2%]) (*P* < .001). Data relating to obstructive shock, a subgroup of other shock, are included in eTable 5 and eTable 6 in the Supplement. In those with cardiogenic shock, prehospital cardiac arrest significantly increased rates of 30-day mortality compared with those that did not have a prehospital cardiac arrest (1061 [69.9%] vs 502 [23.9%]; *P* < .001). Kaplan-Meier survival estimates at 30 days are presented in the Figure. Multivariable analysis for 30-day mortality, adjusted for age and sex, was performed with other causes of shock as the reference group (eTable 7 in the Supplement) (cardiogenic shock: HR, 2.24; 95% CI, 2.05-2.46; septic shock: HR, 1.78; 95% CI, 1.62-1.95; hypovolemic shock: HR, 1.42; 95% CI, 1.26-1.61).

**Table 3.** Outcomes Linked from 30-Day Victorian Death Index and Hospitals

Outcome	Patients by shock etiology, No. (%)					*P* value
	All (N = 12 695)	Cardiogenic shock (N = 3615)	Septic shock (N = 3998)	Hypovolemic shock (N = 1457)	Other causes (N = 3625)	
30-d Mortality	4158 (32.8)	1563 (43.2)	1520 (38.0)	416 (28.6)	659 (18.2)	<.001
Emergency department discharge destination						
Died in department	818 (7.2)	414 (12.9)	169 (4.7)	60 (4.6)	175 (5.2)	<.001
Home	963 (8.4)	157 (4.9)	132 (3.7)	68 (5.2)	606 (18.1)	
ICU	2206 (19.3)	693 (21.5)	746 (21.0)	229 (17.5)	538 (16.0)	
Coronary care	417 (3.6)	254 (7.9)	66 (1.9)	14 (1.1)	83 (2.5)	
Other ward	5612 (49.1)	965 (30.0)	2278 (64.0)	749 (57.3)	1620 (48.3)	
Cardiac catheterization laboratory	544 (4.8)	517 (16.1)	3 (0.1)	2 (0.2)	22 (0.7)	
Operating theater	199 (1.7)	43 (1.3)	19 (0.5)	119 (9.1)	18 (0.5)	
Self-discharge	77 (0.7)	6 (0.2)	7 (0.2)	9 (0.7)	55 (1.6)	
Transfer to other hospital	515 (4.5)	168 (5.2)	138 (3.9)	58 (4.4)	151 (4.5)	
Length of stay, median (IQR), d	2 (1-10)	3 (1-7)	4 (1-8)	2 (1-5)	2 (1-10)	<.001
Time in ICU, median (IQR), h	59 (25-114)	63 (24-121)	63 (31-116)	60 (28-120)	47 (23-85)	<.001
Inpatient mechanical ventilation	2152 (17.0)	1210 (33.5)	335 (8.4)	177 (12.1)	430 (11.9)	<.001
Inpatient dialysis	526 (4.1)	200 (5.5)	148 (3.7)	86 (5.9)	92 (2.5)	<.001
Inpatient coronary angiography	1048 (8.3)	979 (27.1)	21 (0.5)	8 (0.5)	40 (1.1)	<.001
Inpatient PCI	504 (4.0)	493 (13.6)	5 (0.1)	0	6 (0.2)	<.001
Inpatient CABG	23 (0.2)	21 (0.6)	0	1 (0.1)	1 (0.0)	<.001
ECMO support	22 (0.2)	17 (0.5)	2 (0.1)	1 (0.1)	2 (0.1)	<.001
IABP	73 (0.6)	69 (1.9)	1 (0.0)	0	3 (0.1)	<.001

Abbreviations: CABG, coronary artery bypass graft; ECMO, extracorporeal membrane oxygenation; IABP, intra-aortic balloon pump; ICU, intensive care unit; PCI, percutaneous coronary intervention.

**Figure.**
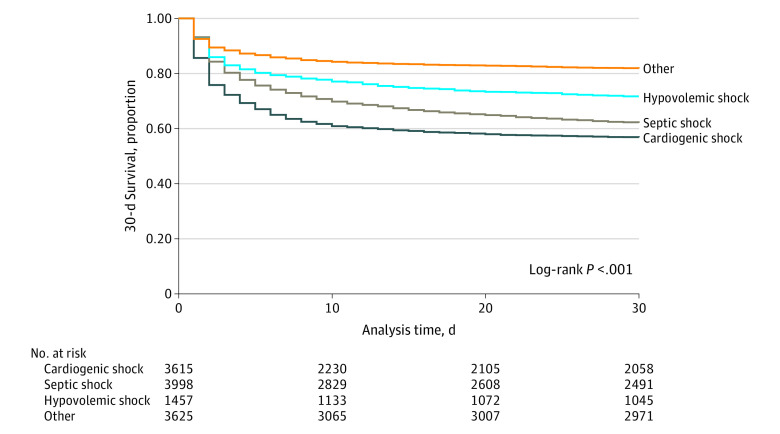
Kaplan-Meier 30-Day Survival Estimates of the Linked Cohort, Stratified by Shock Etiology

Multivariable regression models for the primary outcome of 30-day mortality are presented in Table 4. For the entire matched cohort, increased age (all etiologies: hazard ratio [HR], 1.04; 95% CI, 1.03-1.04), CKD (all etiologies: HR, 1.25; 95% CI, 1.10-1.42), history of heart failure (all etiologies: HR, 1.21; 95% CI, 1.09-1.33), CVA (all etiologies: HR, 1.16; 95% CI, 1.04-1.30), COPD (all etiologies: HR, 1.15; 95% CI, 1.04-1.27), increased initial heart rate (all etiologies: 1.01; 95% CI, 1.00- 1.01), and prehospital intubation (all etiologies: HR, 3.93; 95% CI, 3.48-4.44) were associated with increased risk of 30-day morality. Higher socioeconomic status (all etiologies: HR, 0.96; 95% CI, 0.94-0.98), increased initial systolic blood pressure (all etiologies: HR, 0.99; 95% CI, 0.99-0.99), preexisting coronary artery disease (HR, 0.86; 95% CI, 0.79-0.94), and preexisting hypertension (HR, 0.78; 95% CI, 0.72-0.84) were associated with a lower risk of 30-day mortality. Of note, within the cardiogenic shock group, female sex was strongly associated with an increased risk of 30-day mortality (HR, 1.23; 95% CI, 1.09-1.39).

**Table 4.** Cox Proportional Hazards Models for 30-Day Mortality, by Shock Etiology

Variables	HR (95% CI)^a^				
	All	Cardiogenic shock	Septic shock	Hypovolemic shock	Other
Age	1.04 (1.03-1.04)^b^	1.02 (1.02-1.03)^b^	1.03 (1.03-1.04)^b^	1.04 (1.03-1.05)^b^	1.05 (1.04-1.05)^b^
Female sex	1.00 (0.93-1.07)	1.26 (1.12-1.42)^b^	0.93 (0.83-1.04)	0.84 (0.68-1.05)	0.87 (0.73-1.04)
Prior coronary disease	0.86 (0.79-0.94)^b^	0.93 (0.81-1.06)	0.89 (0.76-1.03)	0.63 (0.46-0.86)^b^	1.04 (0.82-1.31)
Hypertension	0.78 (0.72-0.84)^b^	0.79 (0.70-0.89)^b^	0.76 (0.67-0.86)^b^	0.90 (0.71-1.14)	0.80 (0.66-0.97)^c^
Chronic kidney disease	1.25 (1.10-1.42)^b^	1.20 (0.95-1.50)	1.16 (0.95-1.42)	1.62 (1.13-2.31)^b^	1.38 (0.97-1.96)^d^
Prior heart failure	1.21 (1.09-1.33)	1.28 (1.09-1.51)^b^	1.12 (0.96-1.32)	1.06 (0.75-1.51)	1.42 (1.10-1.84)^b^
CVA	1.16 (1.04-1.30)^c^	1.22 (1.0-1.50)^c^	1.05 (0.87-1.25)	1.41 (1.00-1.99)^d^	1.16 (0.88-1.54)
COPD	1.15 (1.04-1.27)^b^	1.21 (1.02-1.45)^c^	0.87 (0.74-1.02)^d^	1.28 (0.90-1.83)	1.23 (0.94-1.60)
Initial systolic BP	0.99 (0.99-0.99)^b^	0.99 (0.99-1.00)^b^	0.99 (0.99-0.99)^b^	0.99 (0.99-1.00)^b^	0.99 (0.99-0.99)^b^
Initial heart rate	1.01 (1.00-1.01)^c^	1.01 (1.00-1.01)^b^	1.01 (1.00-1.01)^b^	1.01 (1.00-1.01)^b^	1.01 (1.00-1.01)^b^
Prehospital intubation	3.93 (3.48-4.44)^b^	2.49 (1.91-3.24)^b^	2.53 (1.83-3.51)^b^	6.91 (3.56-13.44)^b^	5.62 (4.12-7.65)^b^
Prehospital cardiac arrest^e^	NA	2.41 (1.82-3.19)^b^	NA	NA	NA
Socioeconomic status^f^	0.96 (0.94-0.98)^b^	0.94 (0.90-0.98)^b^	1.00 (0.96-1.04)	0.97 (0.89-1.05)	0.96 (0.90-1.02)

Abbreviations: BP, blood pressure; COPD, chronic obstructive pulmonary disease; CVA, cerebrovascular accident; HR, hazard ratio; NA, not applicable.

^a^ HRs represent time-to-death analysis using a Cox regression model. Individual analyses were performed for each etiology.

^b^ *P* < .01.

^c^ *P* < .05.

^d^ *P* < .10.

^e^ Prehospital cardiac arrest as determined by the Victorian Ambulance Cardiac Arrest registry was included as a covariate in the model for cardiogenic shock.

^f^ Socioeconomic status uses Index of Relative Socioeconomic Disadvantage decile.

## Discussion

In this retrospective population-based cohort study, we assessed the incidence, etiology, and outcomes of patients who received prehospital care by EMS with shock over a 4.5-year period. Through linkage of 5 statewide registries (EMS, ED, hospital admissions, death, and out-of-hospital cardiac arrest), these data provide a unique insight into an EMS-treated shock population that explores prehospital care, hospital determined etiology and treatment, and clinical outcomes, which is lacking in the current literature.^13^ This study has several key findings. First, the population-wide incidence of shock is 76 per 100 000 person-years and is more common in men, older patients, rural settings, and those with a lower socioeconomic status. Second, shock in the prehospital setting is a relatively common clinical problem that EMS manage, complicating nearly 6.8 per 1000 EMS cases. Third, the overall prognosis for patients with shock in the prehospital setting is poor, with 30-day mortality of approximately 33%. Fourth, increased age, female sex (in cardiogenic shock), reduced initial systolic blood pressure, increased initial heart rate, prehospital intubation, socioeconomic disadvantage, preexisting comorbidities that include chronic kidney disease, history of heart failure, stroke and chronic obstructive airways disease were independently associated with 30-day mortality in our cohort.

The inclusion criteria applied in this study sought to capture patients with sustained hypoperfusion as evidenced by prolonged hypotension (sustained systolic blood pressure <90 mm Hg) or the need for epinephrine administration. While a uniformly accepted definition of shock is lacking, we have sought to harmonize our inclusion criteria with that of major clinical trials and societal guidelines.^14^ Landmark trials that include Intra-aortic Balloon Support for Myocardial Infarction With Cardiogenic Shock (IABP-II), Early Revascularization in Acute Myocardial Infarction Complicated by Cardiogenic Shock (SHOCK-II), and the European Society of Cardiology Heart failure guidelines, define shock as prolonged hypotension with a systolic blood pressure of less than 90 mm Hg for 30 minutes or need for inotrope and/or vasopressor, in addition to clinical and biochemical findings that are not practically obtained in the prehospital environment.^5,15,16^ Unlike the current study, previous prehospital data assessing shock have applied an inclusion criteria of an isolated episode of hypotension obtained with a single blood pressure recording.^13,17,18^ Using an isolated blood pressure recording to define shock may capture a population that includes patients with only transient hypoperfusion and may therefore lack generalizability with current trial data and guideline definitions.^13,17,18^

To our knowledge, this is the first contemporary study assessing the population-wide burden of EMS-attended nontraumatic shock in the prehospital setting. These findings have important implications for health policy makers, clinicians, and researchers, given the significant resources required to treat these patients and the associated risk of morbidity and mortality.^19,20^ We have shown that the incidence of prehospital shock within the state of Victoria is 76 per 100 000 person-years. Of note, those residing in nonmetropolitan locations and postcodes with lower socioeconomic status all had increased incidence of EMS attendance for shock. These results may be partially explained by the increased prevalence of coronary artery disease observed in local government areas with increased social disadvantage.^21^ Furthermore, over the study period, there was a significant temporal reduction in overall shock incidence. This improvement is likely because of several factors, including public health initiatives to reduce modifiable risk factors for cardiovascular disease and effective local education campaigns promoting community members to seek early medical attention with the onset of symptoms suggestive of myocardial infarction.^19,22,23^ Additionally, over the study period, prehospital care of ST-elevated myocardial infarction has evolved, with EMS scope of practice expanding to allow for the administration of fibrinolytic therapy in patients with an estimated time greater than 120 minutes to percutaneous reperfusion, which may reduce symptom-to-reperfusion times and potentially contribute to a reduction in cardiogenic shock incidence.^24,25^ Finally, in Victoria, our group has shown a temporal reduction in the adjusted annual incidence of out-of-hospital cardiac arrest due to cardiac etiology, which likely further contributed to the observed reduction in incidence of shock over the study period.^26^

Increased awareness and streamlined systems of care for patients with shock have led to incremental improvements in survival outcomes.^19,27,28,29^ These measures have included health care worker education regarding sepsis recognition and early antimicrobial administration, emergent revascularization in cardiogenic shock, and selective use of mechanical circulatory support. The overall 30-day survival in our cohort was 67.2%. However, these high rates of survival appear to be associated with the low rates of death in the hypovolemic shock (28.6%) and other causes of shock (18.2%) groups. Conversely, our observed rates of 30-day mortality for septic (38.0%) and cardiogenic (43.2%) shock appear to be elevated compared with other registry data. Analysis of patient outcomes from the Surviving Sepsis Campaign has demonstrated that high compliance with sepsis treatment bundles reduced in-hospital death to 29.0% compared with 38.6% in low compliance hospitals.^29^ A similar observation has been noted in the cardiogenic shock population. The National Cardiogenic Shock Initiative (NCSI) has previously published observational data that support a hemodynamic-guided treatment strategy for patients with cardiogenic shock.^28^ In this study, initial management focused on early investigation and treatment in the catheterization laboratory and resulted in a 72% survival to hospital discharge.^28^ However, unlike the current study, the NCSI cohort contained fewer patients with out-of-hospital cardiac arrest (20%), which may account for the higher observed rates of 30-day mortality in the current study given the stepwise increased risk of death seen in the cardiogenic shock group with prehospital cardiac arrest.

The management of cardiogenic shock in our cohort warrants further exploration. It should be noted that only 21.7% of patients with cardiogenic shock underwent inpatient coronary angiography. These low rates of coronary angiography occur in the context of current society guidelines ascribing a class I indication for urgent angiography in cardiogenic shock with a suspected ischemic cause, suggesting that patients are potentially being undertreated and not deriving the maximum potential benefits of aggressive in-hospital cardiac intervention.^30,31^ Additionally, 5.2% of patients with cardiogenic shock required transfer from the initial receiving hospital’s ED to an alternate hospital. This may be the result of EMS transporting patients to inadequately resourced hospitals. The assessment of individual hospital characteristics was not performed in this study, but regionalized systems of care with specialist referral centers in trauma, cardiac arrest, and myocardial infarction have been shown to improve clinical outcomes.^32,33,34,35,36^ Current ambulance guidelines recommend that patients with shock are transferred to the nearest local ED in a hospital with ICU capabilities (excluding cardiac arrest or ST-elevation myocardial infarction, for which invasive cardiology services should be available). The association between outcomes and hospital characteristics that include shock volume, 24-hour invasive cardiology services, the sophistication of available ICU supports, and the capacity to provide acute mechanical circulatory support warrant further exploration, as this may inform ambulance practice with respect to the transfer of patients with shock to specialist centers.

When assessing risk factors for 30-day mortality through multivariable models, socioeconomic disadvantage, defined by the patient’s residential postcode, was associated with an increased risk of mortality in the whole cohort and among patients with cardiogenic shock. It has previously been described that lower socioeconomic status portends worse outcomes after critical illness, in excess of the background effects of age, acuity of illness, and comorbidities.^37,38^ While intrinsic patient factors may contribute to an excess risk of adverse clinical sequalae, our findings could also represent a potential clustering effect associated with variation in local hospital shock management, given that social disadvantage is defined by geographic areas.

Finally, female sex was associated with increased 30-day mortality rates in those with cardiogenic shock (HR, 1.23; 95% CI, 1.09-1.39). In the case of ST-elevation myocardial infarction, it has been shown that female patients present later to hospital, have delays in reperfusion therapy, and ultimately have worse rates of 30-day mortality compared with male patients.^39,40^ We believe that this study’s concerning finding also warrants further exploration to allow for a targeted approach to redress this disparity in outcomes among female patients with cardiogenic shock.

### Limitations

This study has several limitations. First, the study only includes patients with shock diagnosed in the prehospital setting by EMS. Therefore, our study did not capture those who self-presented to hospital or developed shock after arrival to hospital. Second, because of the linkage technique used, 19% of patients were unable to be successfully matched to the VAED and VEMD registries and were therefore not included in our primary analysis. This may partially be explained by patients admitted to private hospitals (7.3%), which do not contribute data to VEMD, and therefore could represent a potential source of selection bias. Additionally, defining shock is a challenge in clinical practice, and there is no universal operationalized definition. However, in this study we have adopted a pragmatic definition of shock evidenced by sustained hypotension or inotrope requirement. The selected definition may also result in an underestimation of the presence of prehospital hypovolemic shock or other shock etiologies that were fluid responsive.

## Conclusions

In this study, shock complicated 6.8 per 1000 EMS cases. It was more common with older age, male sex, in regional settings, and in areas with increased socioeconomic disadvantage. The presence of shock in the prehospital setting portends a high risk of death within 30 days of EMS attendance, in particular among patients with a diagnosis of cardiogenic or septic shock. Further studies are required to establish if variations in hospital characteristics impact clinical outcomes in this patient cohort.

## Notes

Supplement. **eMethods. **Supplementary Methods **eFigure 1. **Cohort Derivation: Study Flowchart **eTable 1. **Reason for Patients Not Transferred to Hospital **eTable 2. **Shock Incidence Stratified by Year **eTable 3. **Sensitivity Analysis: Cardiogenic Shock with VACAR-Determined Prehospital Arrest vs No Prehospital Arrest **eTable 4. **Thirty-Day Mortality Outcomes for Cardiogenic Shock With and Without VACAR-Determined Prehospital Cardiac Arrest **eFigure 2. **Thirty-Day Kaplan-Meier Survival Estimates for Cardiogenic Shock With and Without VACAR-Determined Prehospital Cardiac Arrest **eTable 5. **Obstructive Cause of Shock Etiology **eTable 6. **Unadjusted 30-Day Mortality Including Obstructive Shock **eTable 7. **Thirty-Day Mortality Adjusted by Age and Sex
