# Supplementary material for: Incidence and Outcomes of Nontraumatic Shock in Adults Using Emergency Medical Services in Victoria, Australia
Source: JAMA Netw Open. 2022 Jan 26;5(1):e2145179. doi: 10.1001/jamanetworkopen.2021.45179 (PMC8792885; doi:10.1001/jamanetworkopen.2021.45179)
Supplement: Supplement. — eMethods. Supplementary Methods eFigure 1. Cohort Derivation: Study Flowchart eTable 1. Reason for Patients Not Transferred to Hospital eTable 2. Shock Incidence Stratified by Year eTable 3. Sensitivity Analysis: Cardiogenic Shock with VACAR-Determined Prehospital Arrest vs No Prehospital Arrest eTable 4. Thirty-Day Mortality Outcomes for Cardiogenic Shock With and Without VACAR-Determined Prehospital Cardiac Arrest eFigure 2. Thirty-Day Kaplan-Meier Survival Estimates for Cardiogenic Shock With and Without VACAR-Determined Prehospital Cardiac Arrest eTable 5. Obstructive Cause of Shock Etiology eTable 6. Unadjusted 30-Day Mortality Including Obstructive Shock eTable 7. Thirty-Day Mortality Adjusted by Age and Sex [file jamanetwopen-e2145179-s001.pdf]

Supplemental Online Content

Bloom JE, Andrew E, Dawson LP, et al. Incidence and outcomes of nontraumatic shock in adults using emergency medical services in Victoria, Australia. *JAMA Netw Open.* 2022;5(1):e2145179. doi:10.1001/jamanetworkopen.2021.45179

eMethods. Supplementary Methods

eFigure 1. Cohort Derivation: Study Flowchart

eTable 1. Reason for Patients Not Transferred to Hospital

eTable 2. Shock Incidence Stratified by Year

eTable 3. Sensitivity Analysis: Cardiogenic Shock with VACAR-Determined Prehospital Arrest vs No Prehospital Arrest

eTable 4. Thirty-Day Mortality Outcomes for Cardiogenic Shock With and Without VACAR-Determined Prehospital Cardiac Arrest

eFigure 2. Thirty-Day Kaplan-Meier Survival Estimates for Cardiogenic Shock With and Without VACAR-Determined Prehospital Cardiac Arrest

eTable 5. Obstructive Cause of Shock Etiology

eTable 6. Unadjusted 30-Day Mortality Including Obstructive Shock

eTable 7. Thirty-Day Mortality Adjusted by Age and Sex

This supplemental material has been provided by the authors to give readers additional information about their work.

© 2022 Bloom JE et al. *JAMA Network Open.*

eMethods. Supplementary Methods

I. Dataset linkage processes

For this study, data linkage was performed to combine electronic patient care record data with key Victorian datasets. These included:

1. Victorian Emergency Minimum Dataset: Victorian Department of Health administrative and clinical data related emergency department (ED) presentations at public hospitals in the state. Data is submitted by individual health services and is then subject to validation checks. For this study, EMS patient identifiers were matched with Department of Health identifiers using Dataflux software with deterministic data linkage and fuzzy matching for variables such as names and dates. ED presentations for matched patients were then linked to ambulance cases as follows:
    a. Where the patient was transported to hospital by ambulance, the VEMD arrival time was required to be within one hour of the ambulance ED arrival time.
    b. Where the patient contacted ambulance but was not transported to hospital, the VEMD arrival time was required to be within 48 hours of the emergency call for ambulance. If multiple VEMD records existed within the 48-hour period, the presentation occurring closest in time to the ambulance call was used.
2. Victorian Admitted Episodes Dataset: Victorian Department of Health demographic, clinical and administrative data relating to each admitted episode of care occurring in public and private hospitals, as well as rehabilitation centres, extended care facilities and day procedure centres in the state. For this study, EMS patient identifiers were matched with Department of Health identifiers using Dataflux software with deterministic data linkage and fuzzy matching for variables such as names and dates. For matched patients, individual admitted episodes of care occurring up to 48 hours after the emergency ambulance call were linked to the ambulance patient care record data. Where multiple admitted episodes were recorded within the 48 hours, the episode occurring closest in time to the ambulance call was used.
3. Victorian Death Index: Victorian Department of Health data capturing the date and cause of all deaths in Victoria. For matched patients, death records were then linked to all ambulance contacts occurring in the study period.

II: Study definitions:

Socio-economic status was determined by The Index of Relative Scio-Economic Disadvantage Score (IRSD), a validated measure that ranks individual post-codes into deciles of relative disadvantage. This score is derived from Census data that includes household income, education level, employment status, occupation, housing ownership, and non-English speaking background(2). For this analysis, we divided the IRSD into quintiles, with the 1st quintile being the most disadvantaged (comprising of ISRD deciles 1 and 2) and the 5th quintile being least disadvantaged (IRSD deciles 9 and 10).

Geographic remoteness was determined through the residential area postcode of each event using The Accessibility and Remoteness Index of Australia (ARIA) – a geographic accessibility index that divides Australia into five classes of remoteness (‘Major City’, ‘Inner Regional’, ‘Outer Regional’, ‘Remote’, and ‘Very Remote’) to reflect relative access to services in non-metropolitan Australia(3).

© 2022 Bloom JE et al. *JAMA Network Open.*

Due to low numbers of patients from 'remote' or 'very remote' regions, these groups were combined with the 'outer regional' group for the purposes of this study.

Etiology of shock was defined in accordance with the final discharge diagnosis from hospital using the International Classification of Diseases (ICD) 10 AM codes. The ICD-10 codes used identify patients with cardiogenic shock remains constant across the different ICD-10-AM editions of codes used by hospitals over the study period (i.e. ICD-10-AM: 8th edition in 2014-15; 9th edition in 2015-16 & 2016-17; 10th edition in 2017-18 & 2018-19).

Cardiogenic Shock:

1. Cardiovascular diagnoses: I00-I99
2. ST-elevation myocardial infarction: I210-I213,I220-I229, I256
3. Non-ST elevation myocardial infarction: I214, I219
4. Unstable angina: I200
5. Stable coronary syndromes: I201, I208, I209, I248-I252, I254,I255,I258, I259
6. Atrial fibrillation: I480-I489
7. Supraventricular tachycardia: I471
8. Other arrhythmia: I441, I442, I456, I458- I461, I469, I470, I472, I479, I490-I499
9. Heart failure: I420-I438,I500-I509
10. Myocarditis: I012, I090,I400-I418,I514,
11. Valvular heart disease: I050-I089,I340-I379,I390-I394
12. Other cardiac: I00-I1528 excluding codes categorised above
13. Other vascular: I600-I99 excluding codes categorised above

Septic shock:

1. Respiratory diagnoses: J00-J998
2. Pneumonia: J100, J110, J120-J189, J22
3. Asthma: J450-J46
4. Exacerbation of COPD: J431-J449, J47-J709,J982,J983
5. Other respiratory: J00-J998 excluding codes categorised above
6. Infectious diagnoses: A000-B99
7. Neoplastic diagnoses: C000-D899

Hypovolemic shock:

1. Aortic aneurysm or dissection: I7100-I7103, I711-I719
2. Gastrointestinal diagnoses: K000-K938
3. GORD, gastritis, oesophagitis: K20-K238,K290-K30
4. Peptic ulcer disease: K250-K289
5. Hepatobiliary: K700-K839,K870
6. Pancreatitis: K850-K869,K871
7. Other gastrointestinal: K000-K938 excluding codes categorised above

Other shock:

1. Rheumatological diagnoses: M0000-M99923
2. Mental Health diagnoses: F000-F99
3. Neurological diagnoses: G000-G998
4. Endocrine diagnoses: E000-E899

© 2022 Bloom JE et al. *JAMA Network Open.*

5. Other medical diagnoses: H000-H959, L00-L998, M0000-M99923, N000-N999, O000-O998, P000-Q999, S001-Z999
6. Pericarditis: I010, I092,I241,I300-I328
7. Non-specific pain: R000-R99
8. Pulmonary embolism: I260, I269
9. Pneumothorax: J930-J939

Procedures in Table 3 were defined according to ICD 10 V9.0 procedural codes as follows:

1. Coronary angiography: 3820300, 3820600, 382500, 3821800-3821802
2. Percutaneous coronary intervention: 3830600-3830605, 3831200, 3831201, 3831800, 3831801
3. Coronary artery bypass graft surgery: 3849700-3849707, 3850000-3850005, 3850300-3850305, 9020100-9020103
4. Extracorporeal membrane oxygenation (ECMO): 9022500
5. Intra-aortic balloon pump (IABP): 3836200

© 2022 Bloom JE et al. *JAMA Network Open.*

eFigure 1: Cohort Derivation: Study Flowchart

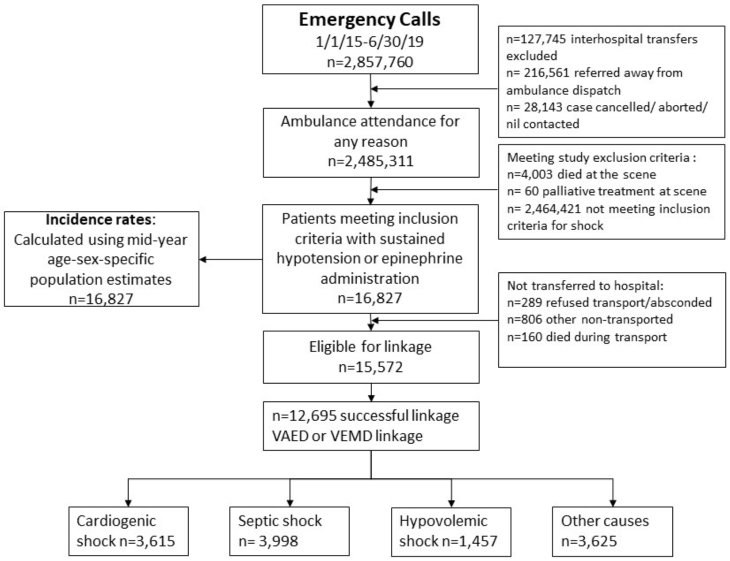

Figure 1: Consort diagram of cohort derivation. VACIS=Victorian Ambulance clinical Information System, VAED=Victorian Admitted Episodes Database, VEMD=Victorian Emergency Minimum Dataset

© 2022 Bloom JE et al. *JAMA Network Open.*

eTable 1: Reason for Patients Not Transferred to Hospital

**Reasons Patient not transferred by Ambulance Crew Logging VACIS case**	
**Reason for not being transported**	**number**
Died at the scene	4003
Bariatric patient	6
Cancelled after arrival	16
Other	543
Transfer not required	167
Patient for palliative management only	60
Referred to local medical officer	43
Referred to other provider	31
Declined transport	280
Patient absconded	9

© 2022 Bloom JE et al. *JAMA Network Open.*

eTable 2. Shock Incidence Stratified by Year

Incidence of ambulance attendances for shock per 100,000 person-years over time											
	2015		2016		2017		2018		2019		
	**Incidence**	**95%CI**	**Incidence**	**95%CI**	**Incidence**	**95%CI**	**Incidence**	**95%CI**	**Incidence**	**95%CI**	**P_{trend}**
Total	94	92-97	84	81-86	66	64-69	65	63-67	67	64-70	<0.001
**Sex**											
Male	96	92-100	86	83-90	68	65-72	69	65-72	73	68-78	<0.001
Female	93	89-97	81	77-84	64	61-67	62	59-65	62	57-66	<0.001
**Age (years)**											
18-29	31	27-34	26	23-29	15	13-18	16	13-18	16	13-20	<0.001
30-39	35	31-39	26	23-29	23	20-27	21	18-24	20	16-24	<0.001
40-49	56	51-62	51	46-56	37	33-41	33	29-37	34	29-40	<0.001
50-59	78	71-84	67	61-73	52	47-57	55	50-60	56	49-64	<0.001
60-69	125	116-134	113	105-122	90	82-97	92	84-99	98	88-109	<0.001
70-79	208	194-223	185	172-199	157	145-169	166	154-178	170	153-188	<0.001
80+	505	477-534	462	436-490	378	354-403	347	325-370	355	323-388	<0.001

Cochran-Armitage test used for p_{trend}
95% confidence interval (95%CI)

© 2022 Bloom JE et al. *JAMA Network Open.*

eTable 3. Sensitivity Analysis: Cardiogenic Shock with VACAR-Determined Prehospital Arrest vs No Prehospital Arrest

	All	Cardiogenic Shock Without Cardiac Arrest	Cardiogenic Shock With Cardiac Arrest	p-value
	N=3,615	N=2,097	N=1,518	
Age	68.3 (16.1)	71.2 (15.9)	64.1 (15.4)	<0.001
Male	2,160 (59.8%)	1,134 (54.1%)	1,026 (67.6%)	<0.001
Accessibility & Remoteness Index of Australia (ARIA)				0.002
Major Cities of Australia	2,600 (73.2%)	1,478 (71.0%)	1,122 (76.3%)	
Inner Regional Australia	783 (22.0%)	494 (23.7%)	289 (19.6%)	
Outer Regional Australia	171 (4.8%)	111 (5.3%)	60 (4.1%)	
Index of Relative Socio-economic Disadvantage (IRSD)				0.026
1	865 (26.8%)	541 (28.5%)	324 (24.3%)	
2	690 (21.4%)	405 (21.3%)	285 (21.4%)	
3	633 (19.6%)	373 (19.7%)	260 (19.5%)	
4	561 (17.4%)	302 (15.9%)	259 (19.4%)	
5	481 (14.9%)	277 (14.6%)	204 (15.3%)	
Hypertension	1,348 (41.5%)	843 (42.1%)	505 (40.6%)	0.41
Dyslipidaemia	841 (25.9%)	537 (26.8%)	304 (24.5%)	0.14
Diabetes	709 (21.8%)	434 (21.7%)	275 (22.1%)	0.76
Coronary artery disease	835 (25.7%)	538 (26.9%)	297 (23.9%)	0.060
Cardiac Failure	439 (13.5%)	317 (15.8%)	122 (9.8%)	<0.001
Chronic Kidney Disease	223 (6.9%)	168 (8.4%)	55 (4.4%)	<0.001
Peripheral Vascular Disease	52 (1.6%)	37 (1.8%)	15 (1.2%)	0.16
Cerebrovascular Disease	240 (7.4%)	166 (8.3%)	74 (6.0%)	0.013
Airways Disease	320 (9.9%)	187 (9.3%)	133 (10.7%)	0.21
Initial Systolic Blood Pressure (mmHg) – median [IQR]	NA	70 [58-80]	0 [0-0]	NA
Initial Heart Rate (BPM) – median [IQR]	84 [50-124]	86 [60-127]	80 [20-120]	<0.001
Pre Hospital Intubation	1,385 (38.3%)	69 (3.3%)	1,316 (86.7%)	<0.001
Adrenaline infusion commenced	1,960 (54.2%)	607 (29.0%)	1,353 (89.1%)	<0.001
Maximum Adrenaline Infusion (mcg/min)	40.9 (57.0)	12.2 (11.7)	53.0 (63.8)	<0.001

* Pre-hospital cardiac arrest determined by Victorian Ambulance Cardiac Arrest Registry (VACAR)
Total numbers and percentages or mean ± standard deviation are presented unless otherwise indicated

© 2022 Bloom JE et al. *JAMA Network Open.*

eTable 4. Thirty-Day Mortality Outcomes for Cardiogenic Shock With and Without VACAR-Determined Prehospital Cardiac Arrest

	All (N=3,615)	Cardiogenic Shock Without Cardiac Arrest (N=2,097)	Cardiogenic Shock With Cardiac Arrest (N=1,518)	p-value
30-Day Mortality	1,563 (43.2%)	502 (23.9%)	1,061 (69.9%)	<0.001
Median length of stay (days) – median [IQR]	3 [1-8]	3 [1-6]	4 [1-9]	<0.001
Median ICU length of stay (hours) – median [IQR]	61 [27-117]	45 [22-94]	71 [26-133]	<0.001

© 2022 Bloom JE et al. *JAMA Network Open.*

eFigure 2. Thirty-Day Kaplan-Meier Survival Estimates for Cardiogenic Shock With and Without VACAR-Determined Prehospital Cardiac Arrest

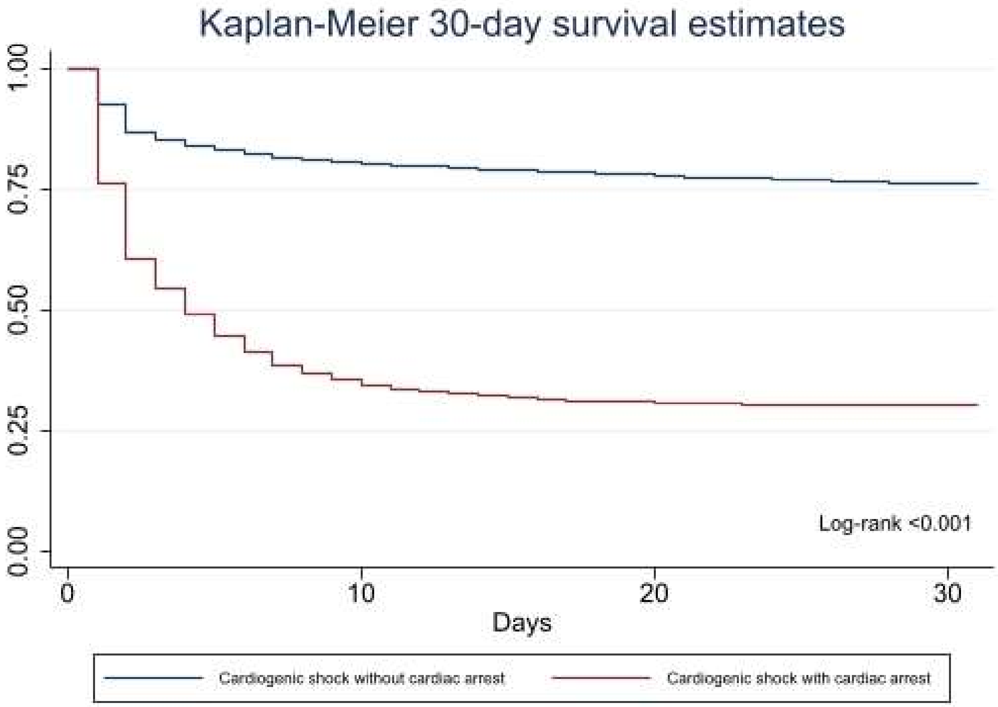

© 2022 Bloom JE et al. *JAMA Network Open.*

eTable 5. Obstructive Cause of Shock Etiology

Presented are patients with an ICD code consistent with obstructive shock – Pneumothorax (ICD 10: J930-J939) , Pericardial disease (ICD 10:I010, I092,I241,I300-I328) and pulmonary embolus (ICD 10: I260, I269). Within the primary analysis, these patients were included as “other” causes of shock:

**Obstructive shock**	
**Cause (n=127)**	**Number**
Pneumothorax	5
Pericardial disease/effusion	17
Pulmonary embolism	105

© 2022 Bloom JE et al. *JAMA Network Open.*

eTable 6. Unadjusted 30-Day Mortality Including Obstructive Shock

**30-day unadjusted mortality**		
	**HR***	**95%CI†**
Cardiogenic shock	2.9	2.5-3.2
Septic Shock	2.3	2.1-2.6
Hypovolemic Shock	1.7	1.5-1.9
Obstructive Shock ‡	2.2	1.6-3.0
Other Shock	REF	REF

*Hazard ratios (HRs) derived using a Cox regression model with cardiogenic shock as the reference
† 95% confidence interval (95%CI)
‡ Obstructive shock has included ICD diagnoses for pulmonary embolus, pneumothorax and pericardial disease/tamponade

© 2022 Bloom JE et al. *JAMA Network Open.*

eTable 7. Thirty-Day Mortality Adjusted by Age and Sex

**30-day mortality adjusted for age and sex**		
	**HR***	**95%CI†**
Cardiogenic shock	2.2	2.0-2.5
Septic Shock	1.8	1.6-2.0
Hypovolemic Shock	1.4	1.3-1.6
Other Shock	REF	REF

*Hazard ratios (HRs) derived using a Cox regression model with Other Shock as the reference
† 95% confidence interval (95%CI)

References:

1. Nehme Z, Bernard S, Cameron P, Bray JE, Meredith IT, Lijovic M, et al. Using a Cardiac Arrest Registry to Measure the Quality of Emergency Medical Service Care. Circ Cardiovasc Qual Outcomes. 2015 Jan 1;8(1):56–66.

2. 2033.0.55.001 seifa 2011 technical paper.pdf [Internet]. [cited 2021 Jul 4]. Available from: https://www.ausstats.abs.gov.au/ausstats/subscriber.nsf/0/22CEDA8038AF7A0DCA257B3B00116E34/$File/2033.0.55.001%20seifa%202011%20technical%20paper.pdf

3. 12160_2006.pdf [Internet]. [cited 2021 Jul 4]. Available from: https://www.ausstats.abs.gov.au/ausstats/subscriber.nsf/0/3E15ACB95DA01A65CA2571AA0018369F/$File/12160_2006.pdf

© 2022 Bloom JE et al. *JAMA Network Open.*
